# *Chlamydia gallinacea,* not *C. psittaci*, is the endemic chlamydial species in chicken (*Gallus gallus*)

**DOI:** 10.1038/srep19638

**Published:** 2016-01-18

**Authors:** Weina Guo, Jing Li, Bernhard Kaltenboeck, Jiansen Gong, Weixing Fan, Chengming Wang

**Affiliations:** 1 Jiangsu Co-innovation Center for Prevention and Control of Important Animal Infectious Diseases and Zoonoses, Yangzhou University College of Veterinary Medicine, Yangzhou, Jiangsu, China; 2 College of Animal Science, Anhui Science and Technology University, Anhui, China; 3 College of Veterinary Medicine, Auburn University, Auburn, Alabama, USA; 4 Poultry Institute, Chinese Academy of Agricultural Sciences, Yangzhou, Jiangsu China; 5 Laboratory of Zoonosis, China Animal Health and Epidemiology Center, Qingdao, Shandong, China

## Abstract

To investigate the prevalence and diversity of *Chlamydia* spp. in domestic birds in China, oral and cloacal swabs of healthy chickens, ducks, geese and pigeons were collected nationwide from live-animal markets and examined by *Chlamydia* spp. 23 S rRNA gene FRET-PCR followed by high-resolution melting curve analysis and confirmatory sequencing. Overall, 26.2% of the birds (602/2,300) were positive for *Chlamydia* spp. and five *Chlamydia* spp. were identified. While occasional detection of *C. suis* and *C. muridarum* in poultry is reported here for the first time, the predominant chlamydial agent was *C*. *gallinacea* representing 63.8% of all positives (384/602) and 81.2% of positive chickens (359/442). Analysis of the *C. gallinacea ompA* phylogeny revealed at least 13 well segregated variants (serovars). Seven-month monitoring of *C. gallinacea*-infected chickens indicated that the infection was persistent. *C. gallinacea*-infected chickens remained without overt clinical disease, but showed body weight gains significantly reduced by 6.5–11.4% beginning in week 3 post-infection. This study indicates that *C. gallinacea* is the endemic chlamydial species in chickens, whereas *C. psittaci* dominates only in pigeons. Further studies are required to address the specific conditions under which *C. gallinacea* could act as an avian pathogen and possibly also a zoonotic agent.

The obligate intracellular bacteria, *Chlamydia* spp., are the etiological agents of chlamydiosis in wild and domestic birds, mammals and humans^1^,^2^,^3^,^4^,^5^. The family *Chlamydiaceae* currently contains the single genus *Chlamydia* (*C.*), which includes 11 recognized species, namely *C. trachomatis*, *C. suis, C. muridarum*, *C. pneumoniae*, *C. abortus*, *C. caviae*, *C. felis*, *C. pecorum*, *C. psittaci*, and two recently introduced species, *C. avium* and *C. gallinacea*^6^.

Until recently, *C. psittaci* was considered to be the only pathogenic chlamydial species in birds, but recent evidence suggested that avian chlamydiosis may also involve *C. gallinacea* and *C. avium*^7^,^8^, *C. abortus*^9^, as well as *C. pecorum* or *C. trachomatis*^10^. Although most avian chlamydial infections remain asymptomatic^11^, they can lead to respiratory, enteric and ocular disease under appropriate conditions^12^,^13^.

So far, *C. gallinacea* has been isolated from chickens, ducks, guinea fowl, turkey and other domestic poultry in four European countries^7^,^14^. This new emerging agent was predominantly found in asymptomatic poultry, but a case of slaughterhouse workers presenting with atypical pneumonia was also reported in association with exposure to *C. gallinacea*-carrying chickens^1^. Both pathogenicity and possible zoonotic potential of *C. gallinacea* have yet to be systematically investigated.

As currently available data on chlamydial infections of domestic birds in China are confined only to three recent reports on *C. psittaci* in poultry^15^,^16^,^17^, this study was undertaken to explore the occurrence of individual chlamydial species in domestic birds, as well as to address the pathogenicity of *C. gallinacea*.

## Results

### Establishment of a FRET-qPCR platform to enable rapid and specific diagnosis for *Chlamydia* spp

**Figure 1:**
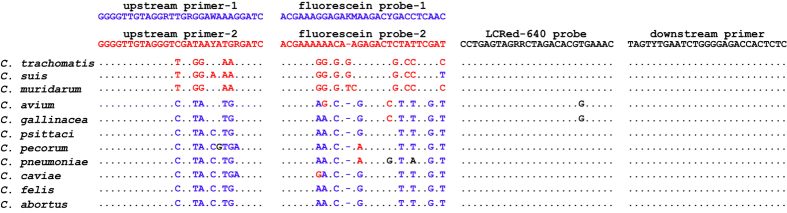
Alignment of primers and probes for 11 *Chlamydia* species. Nucleotide sequences of the primers and probes (two upstream primers, one downstream primer, two fluorescein probes and one LCRed-640 probe) are shown above the corresponding nucleotide sequences of 11 *Chlamydia* species. The LCRed-640 probe and downstream primer show 0 or 1 mismatches with all 11 *Chlamydia* species. Upstream primer-1 and fluorescein probe-1 are specific for *C. trachomatis*, *C. suis* and *C. muridarum*, and mismatches of chlamydial species are indicated by blue or black font. Upstream primer-2 and fluorescein probe-2 are specific for the remaining 8 *Chlamydia* species, and mismatches are indicated by red or black font. The upstream primer and probes were used as shown while the downstream primer was used as reverse complement. Dots indicate that nucleotides are identical to those of primers/probes, and dashes indicate deletion of nucleotides.

**Figure 2:**
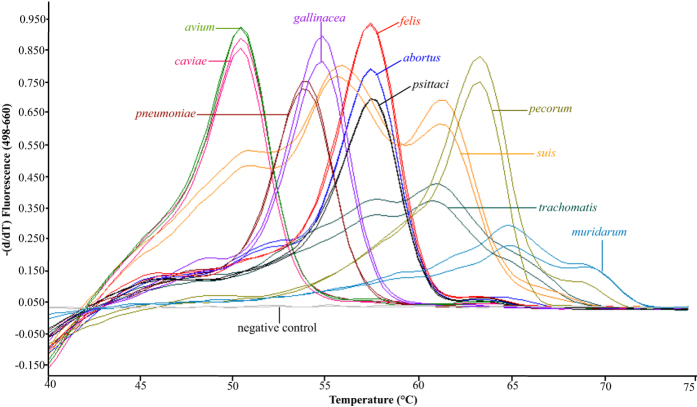
*T*_m_ differentiation of 11 *Chlamydia* species into eight groups through HRM genotyping analysis. Following the completion of PCR, the *T*_m_ of probe hybridization to the targets was determined by high-resolution melting (HRM) curve analysis as the peak of the second derivative of the fluorescence released during a temperature increase from 38–85 °C. Based on the unique *T*_m_ distributions, 11 *Chlamydia* species are differentiated into 8 distinct groups: 50.6 °C for *C. avium* and *C. caviae*; 53.8 °C for *C. pneumoniae*; 54.9 °C for *C. gallinacea*; dual peaks of 55.8 °C and 61.4 °C for *C. suis*, 57.6 °C for *C. felis*, *C. abortus* and *C. psittaci*; a flat peak of 61.0 °C for *C. trachomatis*; 63.3 °C for *C. pecorum*; a flat peak of 65.0 °C for *C. muridarum*. For each *Chlamydia* species, five concentrations of the targets were used (only 100 and 10 copies of the gene/20 μl reaction system are shown here) and peaks and curve shape were consistent at all dilutions.

The 23S rRNA gene-based FRET-qPCR established in this study detects all 11 *Chlamydia* species with high sensitivity and does not give a signal with other related bacteria. Multiple PCRs at 1 copy of chlamydial target per reaction yielded a Poisson distribution of positive and negative amplification reactions, indicating a robust PCR methodology that reliably amplified single target copies. Due to mismatch differences between the probes and the 23S rRNA sequences of 11 *Chlamydia* species (Fig. 1), melting curve analysis of the PCR products enabled us to classify the 11 *Chlamydia* species into 8 distinct groups (Fig. 2). For each *Chlamydia* species, the peaks and shapes of the melting curves were consistent at each of the four concentrations of the targets studied (10,000, 1,000, 100 and 10 copies of 23S rRNA/ 20 μl reaction system) (Fig. 2). A selection of 193 highly *Chlamydia*-positive samples based on FRET-qPCR (above 100 *Chlamydia* genomes/reaction, equivalent to 2,000 genomes/swab) were further verified in confirmatory PCRs targeting highly variable regions of the 16 S rRNA (697 bp) and 23S rRNA (329 bp) genes, and by sequence determination of the amplification products for precise species identification (Table S1).

### PCR prevalence of *Chlamydia* spp. in poultry

The *Chlamydia* FRET-PCR established in this study showed that 26.2% (602/2,300) of apparently healthy chickens, ducks, geese and pigeons from live-animal markets in 24 provinces of China were positive for *Chlamydia* spp. Among 471 *Chlamydia*-positive oral swabs, 372 (79.0%) contained 1–2,000 genomes, and 99 swabs had higher loads up to 447,141 genomes/swab. Concerning cloacal swabs, 209 of 293 *Chlamydia*-positives (71.3%) had 1–2,000 genomes/swab, and 84 swabs contained higher loads up to 966,977 genomes/swab. Overall, pigeons were most frequently infected (49.3%; 106/215), followed by chickens (24.7%; 442/1,791), ducks (24.0%; 43/179) and geese (9.6%; 11/115) (Table 1).

**Figure 3:**
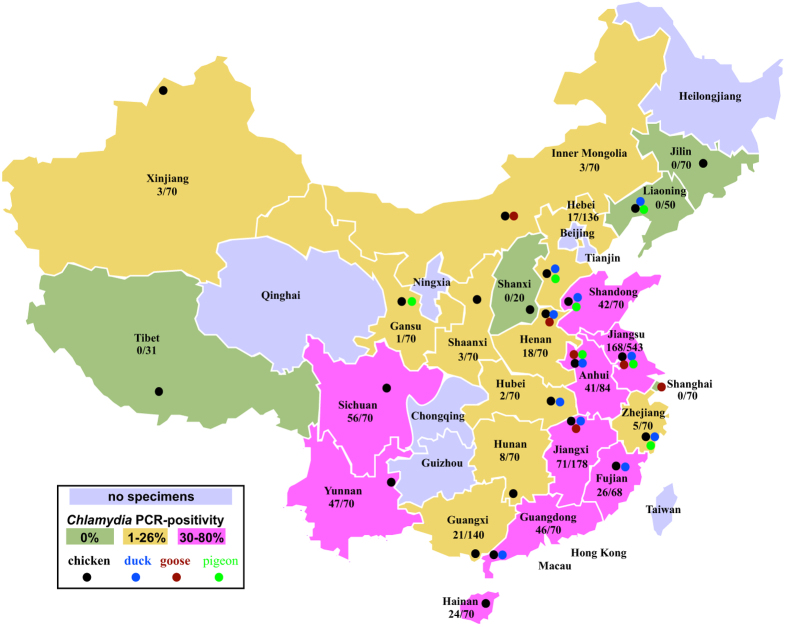
*Chlamydia* spp. prevalence in 24 provinces of China. Overall, 26.2% (602/2,300) of apparently healthy poultry from the live-poultry markets in 24 provinces of China were positive for *Chlamydia* spp. While *Chlamydia* spp. were not detected in 5 provinces (green: Shanxi, Tibet, Jilin, Liaoning and Shanghai), the PCR prevalences were between 1–26% in 10 provinces (yellow: Henan, Guangxi, Hebei, Hunan, Zhejiang, Xinjiang, Shaanxi, Inner Mongolia, Hubei and Gansu), and above 30% in 9 provinces (pink: Sichuan, Yunnan, Guangdong, Shandong, Anhui, Jiangxi, Fujian, Hainan and Jiangsu). Grey color was used to show the provinces where specimens were not available for this study. The colors (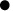 for chicken, 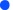 for duck, 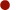 for goose, 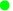 for pigeon) and positions of filled circles indicate different poultry species and the sampling cities. This map was created by YY and CW using Adobe Illustrator CS5 (http://www.adobe.com/products/illustrator.html).

Considerable geographic differences in *Chlamydia* spp. prevalence were found. There was no *Chlamydia* spp. detection in five provinces, while PCR prevalence ranged from 1–26% in ten provinces, and was above 30% in nine other provinces (Fig. 3). Overall, the positivity rate of oral swabs (22.0%; 471/2,138) was significantly higher (*P* < 10^−4^; Chi-square test) than that of cloacal swabs (16.1%; 293/1,824). In birds from which both oral and cloacal swabs were collected (n = 1,662), 162 were positive in both samples while 185 were only positive in oral swabs and 107 only in cloacal swabs. Of the 162 double-positive birds, 142 harbored the same *Chlamydia* species in both swabs.

### Diversity of *Chlamydia* spp. in poultry

The use of FRET-qPCR followed by high-resolution melting curve analysis together with confirmatory PCRs targeting the variable domains of 16S rRNA and 23S rRNA genes enabled us to identify the presence of five *Chlamydia* spp. in the birds examined. The most common species was *C*. *gallinacea* (61.7%; 384/622), followed by *C*. *psittaci* (24.0%; 149/622), *C*. *suis* (10.5%; 65/622), *C. muridarum* (3.4%; 21/622) and *C. pecorum* (0.5%; 3/622) (Table 1). *C*. *gallinacea* and *C*. *psittaci* were the only ones present in all four avian species investigated. The former was detected in 81.2% of the *Chlamydia*-positive chickens (359/442). In contrast, 94.3% of the *Chlamydia*-positive pigeons (100/106) carried *C*. *psittaci* (Table 1).

**Figure 4:**
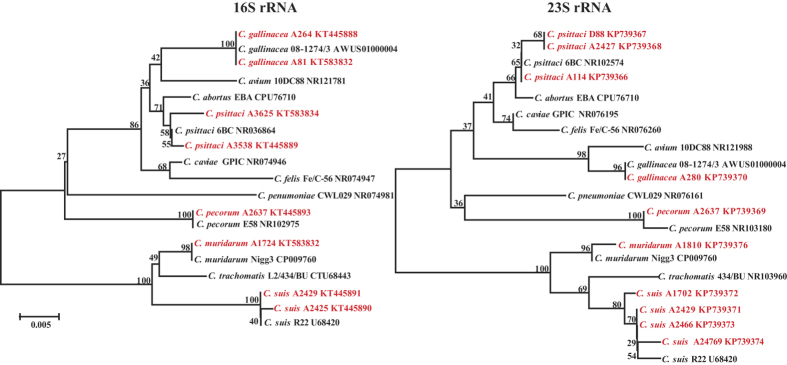
Phylogeny of *Chlamydia* species and strains identified in this study. A 697 bp variable region of the 16S rRNA gene is shown in the left panel, and a 329 bp variable region of the 23S rRNA gene in the right panel. All 11 *Chlamydia* spp. type strains are shown in black font (*Chlamydia* sp, name of strain and sequence accession number), and the *Chlamydia* spp. identified in this study are shown in red font. Branch lengths are measured in nucleotide substitutions and numbers show branching percentages in bootstrap replicates. Scale bar shows the percentage sequence diversity.

Comparative phylogenetic analysis demonstrated that the five *Chlamydia* species identified in this study were all closely related to their respective *Chlamydia* spp. sequences, based on both 16S rRNA (0, 1, 3 mismatches/697 nucleotides; 99.6–100% similarity) (Fig. 4, Table S2) and 23S rRNA genes (0–3 mismatches/329 nucleotides; 99.1–100% similarity) (Fig. 4, Table S3).

### Isolation of *C. gallinacea* strains and *ompA* polymorphism

**Figure 5:**
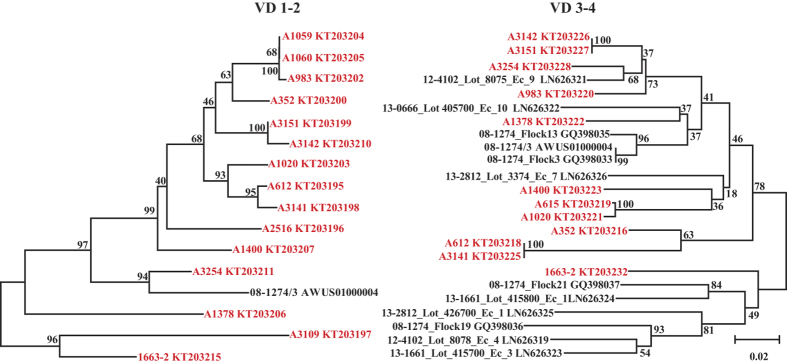
Phylogeny of *ompA* variable domains 1–2 and VD 3–4. A 421 bp region encompassing VD 1–2 is shown on the left panel, and a 435 bp region encompassing VD 3–4 on the right panel. European *C. gallinacea* sequences deposited in GenBank are shown in black font (name of strain and accession number) and strains identified in this study in red font. The inability to amplify every target from every swab specimen resulted in strain differences between the phylograms. Branch lengths are measured in nucleotide substitutions and numbers show branching percentages in bootstrap replicates. Scale bar represents the percentage sequence diversity.

Four *C. gallinacea* isolates (JXC1-4) were obtained from an oral swab and three cloacal swabs of chickens from Jiangxi province by propagation in Hep-2 cells. Their *ompA* sequences (GenBank accession number: KT692977) were identical to each other and showed 85.7% similarity to the only complete *ompA* sequence available in GenBank (strain 08-1274/3; accession number: AWUS01000004). Phylogenetic comparison showed that the *ompA* gene of the *C. gallinacea* strains is highly polymorphic, and at least 13 new variants that were deeply separated, and based on the sequence diversity clearly represent serovars^18^, were identified in addition to the published *ompA* sequences (Fig. 5, Figs S1 and S2).

### Persistent infection of *C. gallinacea* in chickens

**Figure 6:**
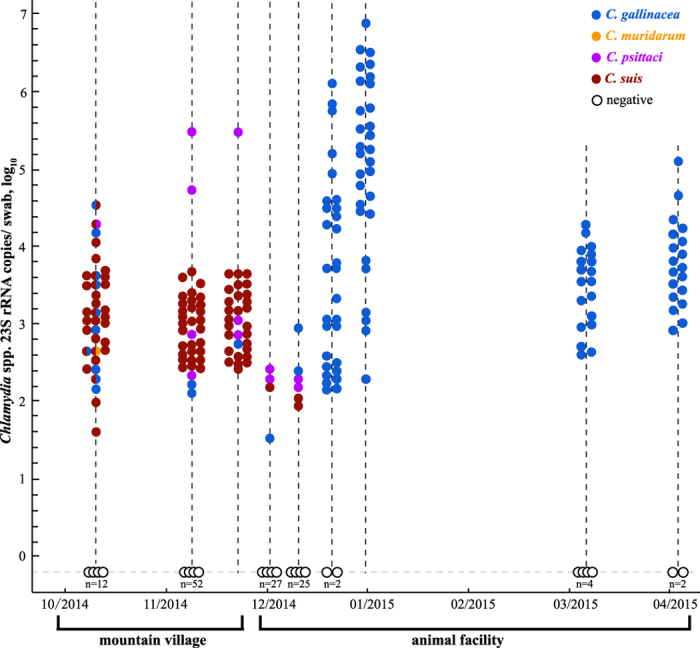
A seven-month monitoring of *Chlamydia* spp. in naturally infected chickens moved from mountain village to animal facility. FRET-qPCR was used to detect DNAs of *Chlamydia* spp. in oral and cloacal swabs of chickens which were moved from a mountain village in Jiangxi province (first three samplings) to a containment animal facility in Jiangsu province (last six samplings). Of the chickens tested in the mountain village, 74.4% (35/47) and 40.9% (36/88) were positive for *Chlamydia* spp. Thirty-one randomly-selected *Chlamydia* spp.-positive chickens (third sampling) were moved to the animal facility on November 27, 2014. Interestingly, four *Chlamydia* species were detected in the first three samplings when the chickens were in mountain village, and the most prevalent species was *C. suis* [31/35 (88.6%), 30/36 (83.3%), 28/31 (90.3%)]. In the first two sampling time points in the animal facility, chlamydial positivity declined precipitously. In the final 4 sampling time points *C. suis* and *C. psittaci* had disappeared from the chickens, but *C. gallinacea* had completely overtaken as the only chlamydial species detected. While no signs of disease were observed, *C. gallinacea*-positivity became very high during the time (29/31, 30/30, 19/23, and 19/21).

Monitoring for *Chlamydia* spp. in naturally-infected chickens in the course of seven months demonstrated persistent *C. gallinacea* infection (Fig. 6), whereas infections with other chlamydial species were observed only transiently. Thirty-one free-range chickens from a mountain village were naturally infected with *C. suis* (28/31; 90.3%), *C. psittaci* (2/31; 6.5%) and *C. gallinacea* (1/31; 3.2%). As shown in Fig. 6, three weeks after transferring these 31 chickens to a containment animal facility, *C. suis* and *C. psittaci* disappeared, while the *C. gallinacea* carrier status was maintained (93.5%; 29/31) until the end of monitoring 3 months later.

In the five euthanized chickens of this group, chlamydial DNA was detected in all cloacal samples, in lung, heart and oral swab (n = 4), liver, trachea, kidney and pancreas (n = 3), and spleen and whole blood (n = 2). The average bacterial genome numbers were highest in cloacal swabs (10^5.15±0.97 [SEM]^/swab), followed by oral swabs (10^3.80±2.62^/swab), lung (10^3.38±1.99^/gram), heart (10^3.35±1.89^/gram), kidney (10^3.07±2.96^/gram), liver (10^2.82±2.61^/gram), pancreas (10^2.61±2.45^/gram), trachea (10^2.51±2.30^/gram), spleen (10^2.00±2.74^/gram) and whole blood (10^1.73±2.45^/ml).

### *C. gallinacea* inoculation of chicken embryos and broiler chickens

To investigate the clinical course and economic impact of *C. gallinacea* infections in chickens, we performed preliminary infection experiments in chicken embryos and chickens. Yolk sac inoculation of one week-old chicken embryos (n = 5) with *C. gallinacea* resulted in 80% mortality of the embryos 8–10 days later (day 8, n = 2; day 9, n = 1; day 10, n = 1). FRET-qPCR determined the highest *C. gallinacea* loads in the yolk sac membrane (2 × 10^7^ genomes/mg), followed by yolk (4 × 10^6^ genomes/mg) and allantoic fluids (2 × 10^6^ genomes/mg). All control chicken embryos (n = 5) inoculated with sucrose-phosphate-glutamate (SPG) buffer only matured normally and hatched as healthy chickens.

**Figure 7:**
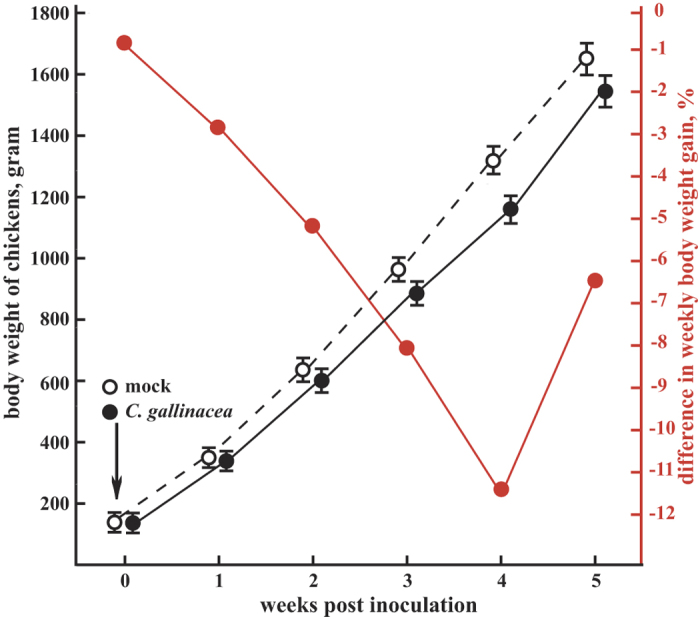
*C. gallinacea* reduces body weight of chickens by up to 11.4%. Body weight (±SEM, left ordinate, in black) is shown for *C. gallinacea*-inoculated AA broiler chickens (filled circle) and mock-inoculated chickens (open circle). The difference in weekly body weight gain is shown in the right ordinate (in red). Compared to mock-inoculated chickens, *C. gallinacea*-inoculated chickens had significantly lower body weights (883 ± 54 [SEM] vs. 962 ± 61 g; 1165 ± 80 vs. 1315 ± 93 g; 1539 ± 120 g vs. 1645 ± 133 g) and lower body weight increase (8.2%, 11.4%, 6.5%, *p* < 0.0009) at 3, 4, and 5 weeks post inoculation.

No signs of clinical disease were observed in *C. gallinacea*-inoculated broiler chickens and mock-inoculated chickens. Body weights did not differ significantly between *C. gallinacea*- and mock-inoculated chickens in the first two weeks pi. However, subsequently in weeks 3–5 pi, *C. gallinacea*-inoculated chickens showed significantly lower body weights than the mock-inoculated chickens. Overall, *C. gallinacea*-inoculated chickens showed 8.2%, 11.4% and 6.5% lower weekly weight gains as compared to the control group in weeks 3–5 pi (Fig. 7).

## Discussion

There are 11 closely related and distinct *Chlamydia* species and most animals are susceptible to multiple *Chlamydia* species, resulting in often asymptomatic infections with low bacterial burdens. While species-specific PCRs have been successfully used to detect individual *Chlamydia* species such as *C. pneumoniae*^19^, *C. trachomatis*^20^,^21^, *C. abortus*^22^, *C. felis*^23^, *C. avium*^24^, *C. gallinacea*^14^, *C. psittaci* and *C. pecorum*^25^, and *C. psittaci* and *C. abortus*^26^, there is a substantial need to establish a genus-specific FRET-qPCR that simultaneously in a single step detects single target copies of all *Chlamydia* spp. and differentiates the amplification product(s) by species. In this study, a novel FRET-qPCR followed immediately by high-resolution melting analysis of the reaction enables the sensitive detection of all chlamydial species and simultaneous differentiation of 11 species into 8 groups. Discriminatory PCRs for sequencing can be performed on *Chlamydia*-positive samples if further differentiation between *Chlamydia* spp. with similar *T*_m_ (between *C. avium* and *C. caviae*; or between *C. felis*, *C. abortus* and *C. psittaci*) is necessary (Fig. 2). This PCR methodology provides a practical and convenient tool for the epidemiological investigation of *Chlamydia* spp. in a variety of sources.

*C. psittaci* has long been considered the main chlamydial species in poultry. While *C. psittaci* was detected in all four avian species investigated in this study, our data showed that *C. psittaci* was the dominant species only in pigeons, representing 94.3% (100/106) of *Chlamydia*-positive pigeons but much lower percentages (9.1–16.3%) in chickens, ducks, or geese (Table 1). The positivity of *Chlamydia* spp. in this study is considerably higher than in other reports. For example, the overall prevalence for *C. psittaci* was 7.9% (26/331) of fecal samples collected from feral pigeons in the Netherlands^27^, and Tanaka *et al.* reported detection of *C. psittaci* in 22.2% (103/463) of feral pigeons in Japan^28^. *C. psittaci* was detected in 18 out of 19 Belgian chicken farms by culture and PCR^29^. Furthermore, it was reported that individuals working in a poultry slaughterhouse with *C. psittaci*-positive ducks presented with cough or flu-like symptoms^30^. Since zoonotic *C. psittaci* was detected in healthy market birds, health workers and consumers should be aware of the possibility of contracting *C. psittaci* infection from such birds, particularly from pigeons.

The results of this study are consistent with the current idea that the epidemiology of avian chlamydiosis is complex and *Chlamydia* infections in birds cannot be automatically ascribed to *C. psittaci*. In agreement with the report from Sachse *et al.*^10^, *C. pecorum* was also detected in chickens in this study. Earlier studies also demonstrated the presence of *C. abortus*, *C. avium* and *C. trachomatis* in birds^8^,^9^,^10^, whereas these species have not been identified in this study. However, we reported for the first time the detection of *C. suis* and *C. muridarum* in chickens, ducks and pigeons. With the exception of the dominance of *C. psittaci* in pigeons, these chlamydial species represented overall sporadic detections, typically confined to “hotspots”. Transmission of these chlamydiae probably occurred when the avian species had close contact to the natural hosts of *C. pecorum*, *C. suis*, or *C. muridarum*.

In contrast, our large and China-wide study involving 2,300 domestic birds demonstrates unambiguously that *C. gallinacea* is the endemic chlamydial species in chickens. The agent was detected in 81.2% of the *Chlamydia*-positive chickens in all four bird species and in 18 of 24 Chinese provinces investigated in this study (Table 1). Phylogenetic analysis of the variable domains of the *OmpA* protein of *C. gallinacea* identified at least 13 well segregated genetic variants (probably serovars), with 10 of them clearly different from known European isolates. This high diversity is consistent with immunoselection of *ompA* variants in endemic infection conditions^31^,^32^. For the time being, poultry must be considered the main natural host for *C. gallinacea.* However, as observed in this study, it is not unusual that *Chlamydia* spp. cross host barriers and infect new hosts or even humans. This may explain that slaughterhouse workers showed atypical pneumoniae after being exposed to *C. gallinacea*-carrying chickens^1^.

The epidemiological survey of *Chlamydia* spp. enabled us to identify a flock of *Chlamydia*-positive chickens in a mountain village that asymptomatically carried *C. suis* and, to a lesser extent, *C. gallinacea, C. psittaci*, and *C*. *muridarum*. Interestingly, three weeks after transfer of 31 birds from that flock to a containment animal facility, *C. suis* and *C. psittaci* completely disappeared while *C. gallinacea* became the most prevalent species. The latter was also detected in blood, oral and cloacal swabs and 8 other organs. This persistent infection with *C. gallinacea* in chickens represents a major epidemiological reservoir. Previous carriage of *C. suis* and *C*. *muridarum* in chickens may have been the result of transient infection from continuous contact to pigs and wild mice in the mountain village.

As evident in the chicken flock from the mountain village, chickens carrying *C. gallinacea* usually do not show clinical signs. To characterize its pathogenic potential, we utilized a *C. gallinacea* isolate obtained in this study to inoculate chicken embryos and SPF broiler chickens and followed the course of infection. While inoculation of chicken embryos resulted in high mortality, intranasal inoculation of 7-day-old broiler chickens with *C. gallinacea* did not generate any clinical signs. However, the infected chickens showed significantly lower body weight gain from 3 weeks pi onwards. This is consistent with the report that asymptomatic endemic *C. pecorum* infections reduce growth rates in calves by up to 48 percent^33^,^34^. In combination with the ubiquitous endemic nature of *C. gallinacea* infection in chickens, this growth-suppressing effect of 10% or more may well be of major economic significance.

In conclusion, by using FRET-qPCR, we have demonstrated that *Chlamydia* spp. are common in healthy domestic birds traded on local live-animal markets in China. Our data confirmed that *C. psittaci* is the dominant species in pigeons, while the new emerging *C. gallinacea* is the endemic species in chickens. This investigation supported the notion that *C. gallinacea* is not a commensal, but a pathogen of moderate pathogenicity, and that persistent infection leads to reduced body weight gain of broilers. It is imperative to further investigate the agent's pathogenicity, host spectrum, transmission mechanisms, and possible zoonotic potential.

## Materials and Methods

### Oral and cloacal swabs

Between December 2013 and February 2014, a total of 3,962 oral and cloacal swabs were collected from 2,300 apparently healthy poultry (1,791 chickens, 215 pigeons, 179 ducks and 115 geese) in live-poultry markets in 24 provinces that encompass all seven geographical regions of China. Sterile cotton swabs were inserted into the throat and cloaca of the chicken and then turned slowly to absorb the fluid sample. All swabs were immediately placed in sterile tubes containing 400 μl DNA/RNA stabilization buffer (Roche Molecular Biochemicals, Indianapolis, IN, USA) and then stored at −80 °C until DNA was extracted as described below. Most of the poultry were free-range raised on family farms, and few animals were originated from industrial poultry operations. Oral and cloacal swabs were obtained from each bird (n =1,662), but in some cases only oral (n = 476) or cloacal swabs (n = 162) were collected. All work in this study was reviewed and approved by the Institutional Animal Care and Use Committee of the Yangzhou University College of Veterinary Medicine (YZUCVM-IACUC 2013#87). The experiments were performed in accordance with the approved IACUC protocols.

### DNA extraction

The High-Pure PCR Template Preparation Kit (Roche Molecular Biochemicals, Indianapolis, IN, USA) was used to extract total nucleic acids from oral and cloacal swabs, the whole blood and organs and tissues from chickens, according to the manufacturer’s instructions and described before^35^. The extracted DNA was eluted in 200 μl elution buffer.

### FRET-qPCR for detection of *Chlamydia* spp

#### Primers and probes

The 23S rRNA sequences for all 11 *Chlamydia* species were obtained from GenBank: *C. pneumoniae* (NR076161, U76711), *C. trachomatis* (NR103960, NR076160), *C. psittaci* (NR102574, JN426968), *C*. *abortus* (NR077001, U76710), *C. pecorum* (NR103180, U68434), *C. muridarum* (NR076163, U68436), *C. suis* (U68420, AF481047), *C. felis* (NR076260, AP006861), *C. caviae* (NR076195, AE015925), *C. avium* (NR121988) and *C. gallinacea* (AWUS01000004). The sequences were aligned using Clustal Multiple Alignment (Vector NTI, Invitrogen, USA) to identify conserved and variable regions suitable for primers and probes that could detect all 11 *Chlamydia* species and largely differentiate the *Chlamydia* species by differential *T*_m_. The master mix of the PCR contained two upstream primers, one downstream primer, two fluorescein probes and one LCRed 640 probe (Fig. 1, Table S1). This PCR amplified a 168 bp fragment of the *Chlamydia* spp. 23S rRNA gene and followed the design described by DeGraves *et al.*^25^, but added an upstream primer and a fluorescein probe.

#### Thermal cycling and melting curve analysis

The FRET-qPCR for *Chlamydia* spp. was performed in a LightCycler 480-II real-time PCR platform using a high-stringency 18-cycle step-down temperature protocol without fluorescence acquisition followed by 30 fluorescence acquisition cycles with a hybridization temperature of 51 °C^25^. The PCR master mix contained two upstream primers (0.5 μM), one downstream primer (1.0 μM) and three probes (0.2 μM). High-resolution melting curve analysis was performed following the completion of the PCR^36^ and data were analyzed as 640 nm: 530 nm (F4/F1) fluorescence ratios with the first derivative of F4/F1 (-d (F4/F1)/dt) evaluated. The fluorescent signals and melting peaks for positive controls, negative controls and other related bacteria were read to determine their positivity.

#### Specificity

To test the specificity of the FRET-PCR established in this study, the DNA of three *Chlamydia* species (*C*. *pneumoniae* strain CDC/CWL-029, ATCC VR-1310; *C*. *trachomatis* serovar D strain UW-3/Cx, ATCC VR-885; *C. psittaci* strain B577, ATCC VR-656) and 8 plasmids manufactured with the pUC57 cloning vector (GenScript, Nanjing, Jiangsu, China) containing an appropriate portion of the 23 S rRNA gene of the remaining 8 *Chlamydia* spp. were used as positive controls. As negative controls, we used DNAs extracted from related bacteria, including *Neisseria sicca*, *Riemerella anatipestifer, Escherichia coli*, *Salmonella* Enteritidis, *Staphylococcus aureus* and *Streptococcus pyogenes* (kindly provided by the Yangzhou University College of Veterinary Medicine). The specificity of the PCR was further verified by electrophoresis of PCR products through 2% agarose gels (BIOWEST®, Hong Kong, China), purification using the QIAquick Gel Purification Kit (Qiagen, Valencia, CA, USA), and sequencing with forward and reverse primers (GenScript, Jiangsu, Nanjing, China).

#### Sensitivity

To test the sensitivity of the FRET-qPCR, PCR amplification products from 11 *Chlamydia* species were gel purified with the QIAquick Gel Extraction Kit (Qiagen, Valencia, CA, USA) and quantified using the PicoGreen DNA fluorescence assay (Molecular Probes, Eugene, OR, USA). To obtain quantitative standards, the molarity of the *Chlamydia* DNA was determined using the calculated molecular mass of the PCR products, which then were adjusted to provide solutions containing 10,000, 1,000, 100, 10, 1 gene copies per PCR reaction in T_10_E_0.1_ buffer as described previously^37^.

### Confirmatory sequencing

*Chlamydia*-positive samples based on FRET-qPCR and high-resolution melting curve analysis were further amplified for DNA sequencing to confirm the identified *Chlamydia* spp. PCRs were designed to target a variable region of 16S rRNA gene (697 bp) and a highly variable region of 23S rRNA gene (329 bp) for all 11 *Chlamydia* spp. (Table S1). To investigate the polymorphism of *ompA* gene in the *C. gallinacea* isolates and clinical specimens, the *ompA* PCR-1 was designed to amplify the complete *ompA* gene of *C. gallinacea* (1,188 bp) while *ompA* PCR-2 and *ompA* PCR-3 amplify the regions of variable domains (VD) 1–2 (435 bp) and VD 3–4 (421 bp) of *C. gallinacea* (Table S1). These PCRs were performed as described above for FRET-qPCR. The PCR products were electrophoresed through 2% agarose gel (BIOWEST®, Hong Kong, China) and purified for automated DNA sequencing (GenScript, Jiangsu, Nanjing, China).

### Isolation of *C. gallinacea*

Human Epidermoid Carcinoma-2 cells (Hep-2 cells) were grown in Iscove’s Modified Dulbecco’s Medium (IMDM, Life Technology, USA) with 10% fetal bovine serum (Life Technology, USA) and amphotericin B (250 μg/ml, Amresco, USA) at 37 °C with 5% CO_2_ in a humidified cabinet for 24–48 h. Swabs in 200 μl SPG buffer from animals that were *C. gallinacea*-positive were transferred for chlamydial isolation into sterile tubes and vortexed with sterile magnetic beads for 3 min. After centrifugation at 1,250 g for 5 min, supernatants were transferred for incubation into 25 cm^2^ cell culture flasks with 7.6 ml IMDM and 0.4 ml antibiotics dissolved in IMDM (vancomycin 100 μg/ml, streptomycin 100 μg/ml, kanamycin 100 μg/ml, amphotericin B 3.75 μg/ml and gentamycin 10 μg/ml).

### Monitoring of *Chlamydia* spp. naturally-infected chickens

Thirty-one locally-bred, free-range chickens, around 42 weeks of age, were transported from a mountain village in Jiangxi Province to a containment animal facility at the Poultry Institute in Jiangsu province. This mountain village has several swine farms and many free-range chickens. The chickens appeared healthy on arrival and were housed on the floor with free access to antibiotics-free food and water. Oral and cloacal swabs were collected aseptically and placed into tubes with 600 μl SPG buffer for isolation of *Chlamydia* spp. and for DNA extraction. Two months after being moved into the animal facility, five randomly-selected chickens were euthanized to test for *Chlamydia* DNA in their blood samples, oral and cloacal swabs, and organs (tracheas, heart, liver, spleen, lung, kidney and pancreas) by FRET-qPCR.

### Chicken embryos, broilers and *C. gallinacea* infection

White Leghorn chicken embryos purchased from Beijing Merial Vital Laboratory Animal Technology Co., Ltd (Beijing, China) were incubated with rotation at 37.8 °C and 60% relative humidity. The yolk sac was inoculated at embryonic development day 7 with 200 μl *C. gallinacea* suspension (2 × 10^6^ genomes) or SPG buffer (as mock inoculation) as described^38^,^39^. Candling was performed once daily to determine the vitality of the chicken embryos.

One-day-old SPF AA broiler chickens obtained from Sandeli Animal Husbandry Development Co., Ltd (Zhenjiang, China) were individually tagged and housed in a containment level 2 facilities with free access to antibiotics-free food and water. After one week, the chickens were separated into two groups that were inoculated intranasally with 20 μl of 2 × 10^6^ genomes of *C. gallinacea* diluted in SPG buffer (n = 15) or an equal volume of SPG buffer as control group (n = 15). The body weight of each chicken was recorded weekly before the inoculation and up to 5 weeks post inoculation while the oral and cloacal swabs were collected to detect the *Chlamydia* DNAs by FRET-qPCR.

### Statistical analysis

Comparisons of the PCR prevalences of *Chlamydia* spp. in oral and cloacal swabs were analyzed by the Chi-Square Test. The two-tailed Tukey honest significant difference (HSD) test (Statistica, StatSoft, Tulsa, USA) was performed to compare the means of the body weight in mock- and *C. gallinacea*-inoculated chickens. Differences at P ≤ 0.05 were considered significant.

## Additional Information

**How to cite this article**: Guo, W. *et al.* *Chlamydia gallinacea*, not *C. psittaci*, is the endemic chlamydial species in chicken (*Gallus gallus*). *Sci. Rep.* **6**, 19638; doi: 10.1038/srep19638 (2016).

## Supplementary Material

Supplementary Information

## Figures and Tables

**Table 1:** *Chlamydia* spp. in poultry from 24 provinces of China.

Provinces	Chicken (442/1791, 24.7%)						Duck (43/179, 24.0%)					Goose (11/115, 9.6%)					Pigeon (106/215, 49.3%)		
	neg*	gal*	*mur*	*psi*	*sui*	*pec*	neg	*gal*	*mur*	*psi*	*sui*	neg	*gal*	*mur*	*psi*	*sui*	neg	*gal*	*psi*
Sichuan	14	56																	
Yunnan	23	47		1															
Guangdong	22	44					2	2											
Shandong	20	39						3									8		
Jiangxi	67	13	1	3	45		37	4	4		3	3	1			6			
Fujian	23	9		1	2		19		10	2	3								
Hainan	46	22			3														
Jiangsu	255	27	2	32	1		36	1	2	4	1	19	1		1	1	65	6	97
Henan	41	13		2			5	1		1		6		1					
Guangxi	109	21					10												
Hebei	101	15					3										15	1	1
Hunan	62	7	1																
Zhejiang	53	4					11	1									1		
Inner Mongolia	62					3						5							
Xinjiang	67	3																	
Shaanxi	67	2		1															
Hubei	62	1		1			6												
Gansu	56	1															13		
Jilin	70																		
Liaoning	37						7										6		
Tibet	31																		
Shanxi	20																		
Anhui	41	35						3				1	1				1		2
Shanghai												70							

^*^gal = *C. gallinacea; mur* = *C. muridarum*; *psi* = *C. psittaci*; *sui* = *C. suis*; *pec* = *C. pecorum*; neg = chlamydial DNA was not detected.
