# Supplementary material for: Chlamydia gallinacea, not C. psittaci, is the endemic chlamydial species in chicken (Gallus gallus)
Source: Sci Rep. 2016 Jan 18;6:19638. doi: 10.1038/srep19638 (PMC4726042; doi:10.1038/srep19638)
Supplement: Supplementary Information [file srep19638-s1.pdf]

Supplementary Information

Chlamydia gallinacea is the endemic chlamydial species in chicken (*Gallus gallus*)

Weina Guo[1,2*], Jing Li[1*], Bernhard Kaltenboeck[3], Jiansen Gong[4], Weixing Fan[5] & Chengming Wang[1]**

Table S1. Oligonucleotide primers and probes used in this study.

**PCR**	**Primer /probe**	**Sequence (5’-3’)**	**Target, length**
FRET-qPCR	UP1	GGGGTTGTAGGRTTGRGGAWAAAGGATC	11 *Chlamydia* spp., 168 bp
	UP2	GGGGTTGTAGGGTCGATAAYATGRGATC	
	DN	GAGAGTGGTCTCCCCAGATTCARACTA	
	FLU1	ACGAAAGGAGAKMAAGACYGACCTCAAC-(6-FAM)	
	FLU2	ACGAAAAAACAAGAGACTCTATTCGAT-(6-FAM)	
	LCRed	LCRed 640-CCTGAGTAGRRCTAGACACGTGAAAC-(Phos)	
16S rRNA PCR	UP	GGGATCTTCGGACCTTTCGGTT	11 *Chlamydia* spp., 697 bp
	DN	TGCTTCTTTACCTGGTACGCTCAAATC	
23S rRNA PCR	UP	GAGTCCGGGAGATAGACAGC	11 *Chlamydia* spp., 329 bp
	DN	CATGGATCTTCACTAGTATCCGC	
ompA PCR-1, whole gene	UP	ATGAAAAAACTCTTGAAATCGGCATTGTT	*C. gallinacea*, 1188 bp
	DN	TTAGAATCTGAATTGAGCATTGACGTGAG	
ompA PCR-2, VD 1-2	UP	TTCGTGCAGGATTCTACGGAGATTATGT	*C. gallinacea*, 435 bp
	DN	AGCACCAATACTCCAGGAGAACGAAGT	
ompA PCR-3, VD 3-4	UP	ATGTTCTGTGTACTCCTGCGCAATTCA	*C. gallinacea*, 421 bp
	DN	TTATCAGCGTCAACTAAAGTGGCTCCA	

* UP and DN stand for upstream or downstream primers; FLU1 and FLU2 are for fluorescent probes while LCRed is for LCRed640 probe; Phos indiciates 3’ phosphorylation for prevention of probe extension.

Table S2. Comparison of isolates identified in this study and similar sequences in GenBank based on 16S rRNA.

***Chlamydia* spp.**	**Isolates identified in this study**		**Best matches in GenBank**		
	GenBank accession	Strain/origin	GenBank accession	Strain/origin	Mismatch
C. psittaci	KT445889	JSO-A3538, from oral swab of pigeon in Jiangsu [Jiangsu(n=8), Jiangxi (n=2), Anhui (n=6)]	NR036864	*C. psittaci* 6BC, from a parakeet in USA	1/697
	KT583834	JSC-A3625, from cloacal swab of duck in Jiangsu [Jiangsu (n=4)]			3/697
C. pecorum	KT445893	IMO-A2637, from oral swab of chicken in Inner Mongolia [Inner Mongolia (n=3)]	NR102975	*C. pecorum* E58, from brain of a calf in USA	0/697
C. gallinacea	KT445888	SCO-A264, from oral swab of chicken in Sichuan [Sichuan (n=3), Jiangsu (n=8), Jiangxi (n=2)]	AWUS010 00004	*C. gallinacea* 08-1274/3, from cloacal swab of *Gallus gallus* in France	0/697
	KT583832	JSC-A81, from cloacal swab of pigeon in Jiangsu [Jiangsu (n=6), Xinjiang (n=2)]			0/697
C. suis	KT445890	JXC-A2425, from cloacal swab of chicken in Jiangxi [Jiangxi (n=4), Fujian (n=2)]	U68420	*C. suis* R22, from *Sus scrofa* in USA	1/697
	KT445891	JXC-A2429, from cloacal swab of chicken in Jiangxi [Jiangxi (n=2)]			0/697
C. muridarum	KT583833	FJO-A1724, from oral swab of duck in Fujian [Fujian (n=2), Jiangxi (n=3)]	CP009760	*C. muridarum* Nigg3, from *Mus musculus* in USA	0/697

Table S3. Comparison of isolates identified in this study and similar sequences in GenBank based on 23S rRNA.

***Chlamydia* spp.**	**Isolates identified in this study**		**Best matches in GenBank**		
	GenBank accession	Strain/origin	GenBank accession	Strain/origin	Mismatch
C. psittaci	KP739366	JSO-A114, from oral swab of duck in Jiangsu [Jiangsu (n=10), Fujian (n=1)]	NR102574	*C. psittaci* 6BC, from a parakeet in USA	0/329
	KP739367	JSO-D88, from oral swab of pigeon in Jiangsu [Jiangsu (n=18), Anhui (n=3)]			1/329
	KP739368	JXC-A2427, from cloacal swab of chicken in Jiangxi [Jiangxi (n=2), Fujian (n=1)]			1/329
C. pecorum	KP739369	IMO-A2637, from oral swab of chicken in Inner Mongolia[Inner Mongolia (n=3)]	NR103180	*C. pecorum* E58, from brain of a calf in USA	1/329
C. gallinacea	KP739370	SCO-A280, from oral swab of chicken in Sichuan [Sichuan (n=6), Jiangsu (n=16), Guangdong (n=12), Xinjiang (n=2)]	AWUS010 00004	*C. gallinacea* 08-1274/3, from cloacal swab of *Gallus gallus* in France	0/329
C. suis	KP739371	JXC-A2429, from cloacal swab of chicken in Jiangxi [Jiangxi (n=11)]	U68420	*C. suis* R22, from *Sus scrofa* in USA	2/329
	KP739372	FJO-A1702, from oral swab of chicken in Fujian [Fujian (n=1), Jiangxi (n=2)]			3/329
	KP739373	JXC-A2466, from cloacal swab of goose in Jiangxi [Jiangxi (n=1)]			1/329
	KP739374	JXC-A2469, from cloacal swab of goose in Jiangxi [Jiangxi (n=1)]			2/329
C. muridarum	KP739376	FJC-A1810, from cloacal swab of duck in Fujian [Fujian (n=3), Jiangxi (n=3), Jiangsu(n=2)]	CP009760	*C. muridarum* Nigg3, from *Mus musculus* in USA	1/329

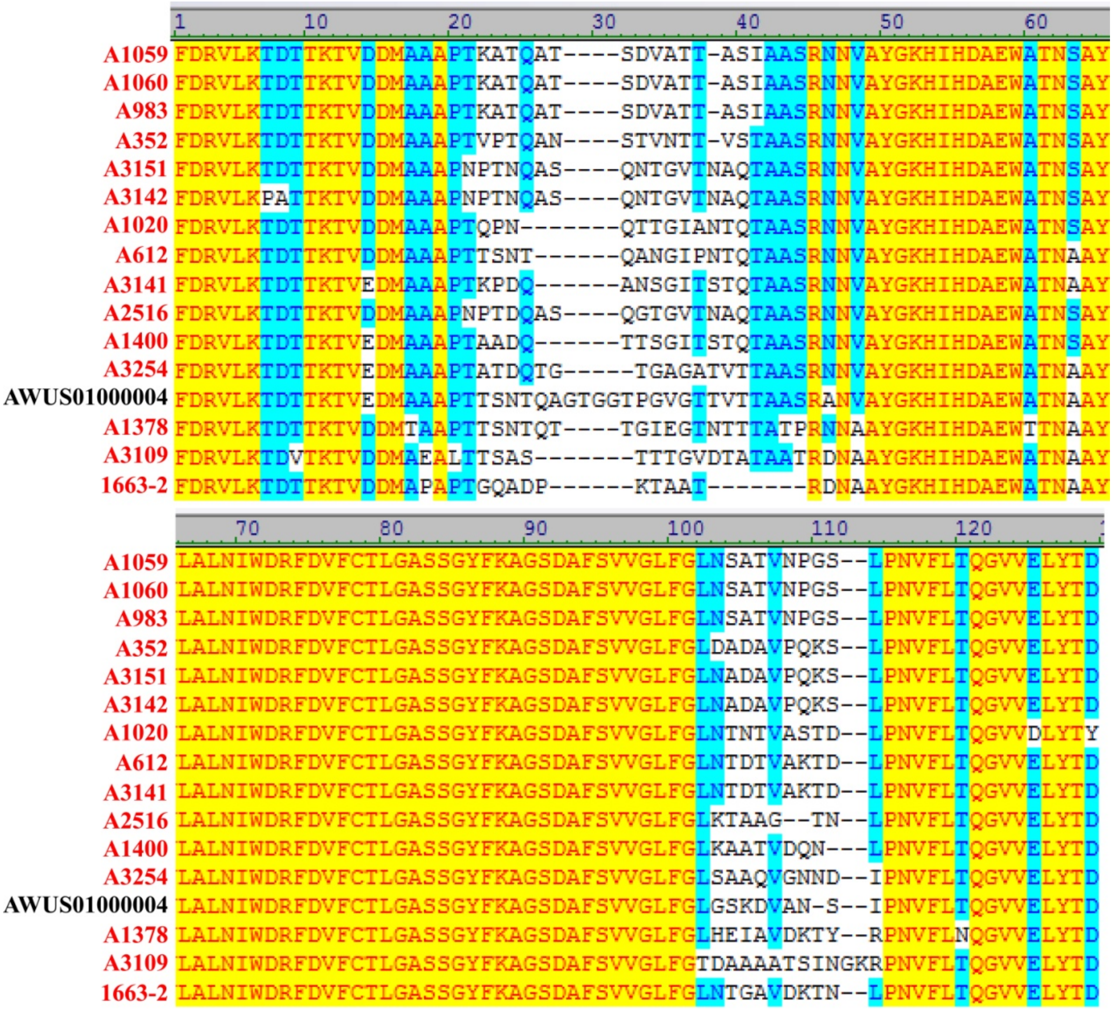

Figure S1. Alignment of 129-amino acid sequences of *ompA* variable domains 1-2. Sequences as shown in the left panel of Figure 5 were translated and pairwise aligned. European *C. gallinacea* sequences deposited in GenBank are shown in black font and strains identified in this study are in red font.

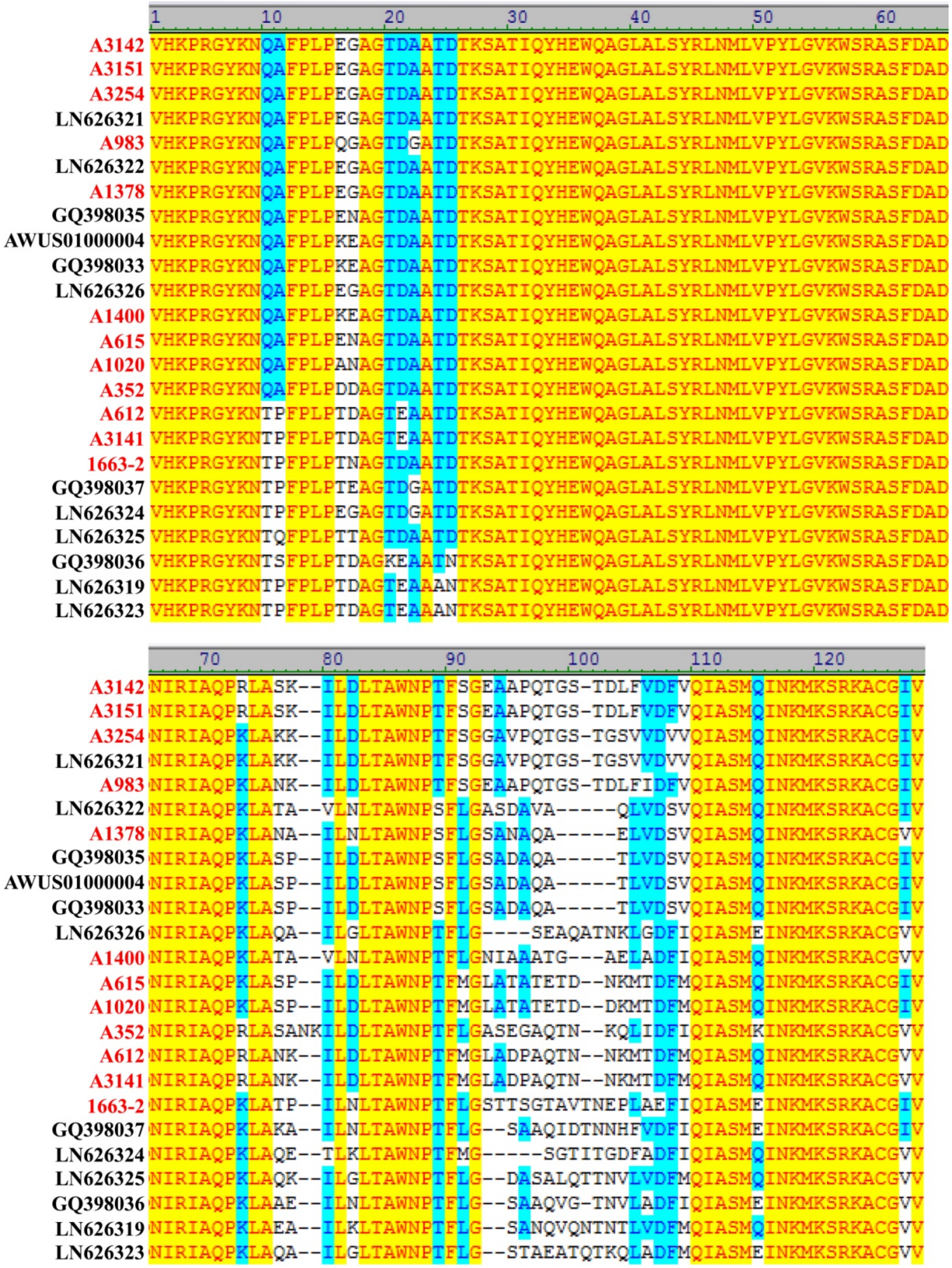

Figure S2. Figure S1. Alignment of 128-amino acid sequences of *ompA* variable domains 3-4. Sequences as shown in the right panel of Figure 5 were translated and pairwise aligned. European *C. gallinacea* sequences deposited in GenBank are shown in black font and strains identified in this study are in red font.
